# Viral Vectors as Gene Therapy Agents for Treatment of Glioblastoma

**DOI:** 10.3390/cancers12123724

**Published:** 2020-12-11

**Authors:** Oleg Mozhei, Anja G. Teschemacher, Sergey Kasparov

**Affiliations:** 1 School of Life Sciences, Immanuel Kant Baltic Federal University, 236041 Kaliningrad, Russia; 2 School of Physiology, Neuroscience and Pharmacology, University of Bristol, Bristol BS8 1TD, UK; anja.teschemacher@bristol.ac.uk

**Keywords:** gene therapy, glioblastoma, glioma, viral vectors

## Abstract

**Simple Summary:**

Glioblastoma is the most malignant cancer of the brain and current therapeutic strategies are clearly inadequate. In addition to surgical intervention, conventional drugs and ratio-therapy, scientists are looking at approaches based on gene therapy with genetically modified viruses. In this review we give a snapshot of the current state of play in this field of research and the available information about the clinical trials. We make some suggestions as to what opportunities could be explored further and hope that this review will stimulate discussion and conception of new life saving strategies.

**Abstract:**

In this review, we scrutinize the idea of using viral vectors either as cytotoxic agents or gene delivery tools for treatment of glioblastoma multiforme (GBM) in light of the experience that our laboratory has accumulated over ~20 years when using similar vectors in experimental neuroscience. We review molecular strategies and current clinical trials and argue that approaches which are based on targeting a specific biochemical pathway or a characteristic mutation are inherently prone to failure because of the high genomic instability and clonal selection characteristics of GBM. For the same reasons, attempts to develop a viral system which selectively transduces only GBM cells are also unlikely to be universally successful. One of the common gene therapy approaches is to use cytotoxic viruses which replicate and cause preferential lysis of the GBM cells. This strategy, in addition to its reliance on the specific biochemical makeup of the GBM cells, bears a risk of necrotic cell death accompanied by release of large quantities of pro-inflammatory molecules. On the other hand, engaging the immune system in the anti-GBM response seems to be a potential avenue to explore further. We suggest that a plausible strategy is to focus on viral vectors which efficiently transduce brain cells via a non-selective, ubiquitous mechanism and which target (ideally irreversibly) processes that are critical only for dividing tumor cells and are dispensable for quiescent brain cells.

## 1. Introduction

Glioblastoma multiforme (GBM) is a highly malignant primary brain cancer of predominantly astrocytic origin [1]. The main features of GBM that lead to malignancy and high mortality are its high resistance to DNA-damaging drugs, including the only Food and Drug Administration FDA-approved alkylating agent temozolomide (TMZ), which is achieved by O6-methylguanine-DNA methyltransferase overexpression, moderate response to radiation, genomic instability and powerful clonal selection. A particularly grave feature of GBM is its high invasiveness.

New insights into the genomic landscape of GBM revealed typical mutations in an array of genes, including *TERT*, *PTEN*, *IDH1*, *IDH2*, *TP53*, *ATRX*, *PIK3CA*, *PIK3R1*, *NF1*, *H3F3A*, *CDKN2A*, *EGFR*, *PDGFRA*, *MET*, *CDK4*, *CDK6*, *MDM2*, *MDM4* [2]. Traditionally, based largely on neuroanatomical considerations, gliomas were subdivided into four grades. Glioblastoma is the most malignant (grade IV) glioma [3].

The introduction by the World Health Organisation (WHO), in 2016, of the “integrated” classification based on histology and genetics was developed in the hope of improving diagnostic accuracy, patient management and prognosis of the response to treatments [4]. However, as of today, most of the treatment algorithms are not based on molecular histological characteristics and are essentially universal, consisting of maximal surgical resection, followed by radiotherapy and chemotherapy with TMZ, followed by TMZ, known as the “Stupp protocol” [5,6].

Unfortunately, even this aggressive treatment has low efficiency, with survival rates remaining between 12 and 15 months and the 3-year survival rate only at about 15%. Despite introduction of newer treatments, such as Carmustine wafers, the monoclonal antibody bevacizumab and cyclin-dependent kinases (CDK) inhibitors, GBM is still an essentially incurable disease, resulting in a patient death rate of more than 95% within five years of diagnosis.

Even though classic metastases are exceedingly rare in GBM, its cells have a tendency to migrate into the parenchyma and eventually spread extensively throughout the brain. For this reason, already upon primary diagnosis, some patients have infiltration in more than one part of the brain, with tumor cells moving across the corpus callosum or through the walls of the ventricles. In cases such as those, surgery may be performed only for the sake of decompression but has little effect on the overall progression of the disease. The only feasible option to pursue, then, is systemic pharmacotherapy and radiotherapy. However, GBM presents formidable challenges for traditional drug design. Movement of drugs across the blood–brain barrier (BBB) is a significant problem because it depends on too many factors (charge, molecular weight and conformation, hydrophobicity, presence of specific transporters, vascularization of the tumor, etc.). Moreover, the relationship between these factors and drug transfer across the BBB is non-linear. It is estimated that less than 2% of small-molecule drugs and no large-molecule drugs or nucleic acid-based constructs can reach the brain because of the BBB [7]. Insufficient saturation of brain tissue with anti-cancer drugs allows GBM cells to benefit from the selection of the most aggressive and drug-resistant subclones. In addition, tumors engage various efflux transport systems (for instance, ATP-binding cassette sub-family B member 1 (*ABCB1*) gene, which extrude drugs from cancer cells) [8]. The other well-known mechanism of tumor defense is expression of high levels of the DNA repair enzyme O6-methylguanine-DNA methyltransferase, mentioned above [9].

However, upon initial diagnosis, GBM tumors frequently appear relatively well-localized and surgically accessible. Nevertheless, due to the infiltration, tumors almost inevitably reoccur after resection, typically originating from sites adjacent to the surgical cavity. Surgeons are limited in their actions because GBM often grows near critical regions of the brain (major nerve tracts, essential centers and large blood vessels). Damage to those areas is too risky and may cause severe disabilities or even be lethal. In cases of well-localized and relatively superficial primary GBM, the key task is, therefore, the prevention of infiltration around the surgical cavity. Here is the scope for locally delivered therapies, such as slow-release formulations of anti-cancer drugs [10], photodynamic therapy [11] or viral vectors, which are the topic of this review.

## 2. Molecular Strategies for Viral Gene Therapy of the GBM

For patients with well-localized primary GBM, one could envisage a strategy where after the de-bulking surgery, the adjacent parenchyma is infiltrated by viral gene therapy vectors which selectively destroy the GBM cells. In a more dramatic scenario, a viral gene therapy tool could be injected systemically, selectively affecting tumor cells in the whole of the CNS and eliminate them. Attempts to develop gene therapy with the aid of viral vectors have been under development for some time, and below, we summarize some of the main strategies and their outcomes.

1. Oncolytic viruses which destroy tumor cells were amongst the first vectors which were tested in patients. The rationale for this approach was based on pre-clinical data demonstrating that some strains of various viruses replicate well only in tumor cell lines. It was then suggested that it is possible to selectively destroy cancer cells in situ, with minimal impact on normal cells. In clinical studies, either wild-type or genetically engineered viruses were used; the specificity of the latter was enhanced by targeted changes in their genomes. It needs to be stressed that oncolytic viruses are able to destroy any cells which they invade and, unless tightly controlled by an additional mechanism, might cause excessive tumor necrosis and dangerous brain oedema [12,13]. While several viral progenitors have been used (see Section 2.1 below), the first oncolytic viruses were wild-type viruses, followed by second generations of genetically modified viruses and third-generation vectors equipped with transgenes to further induce therapeutic effects [12].

2. Suicide gene therapy is based on heterologous expression of *Escherichia coli* or yeast cytosine deaminase or *Herpes simplex* virus thymidine kinase in the cancer cells [14]. Cytosine deaminase converts the prodrug 5-Fluorocytosine (5-FC) to a toxic 5-Fluorouracil (5-FU) metabolite, whereas thymidine kinase (HSV-tk) converts ganciclovir to ganciclovir monophosphate, which, in turn, is converted to toxic ganciclovir triphosphate by tumor cells’ enzymes. This leads to damage and lysis of transgene-expressing cells and those surrounding them (so-called bystander effect).

3. Immunomodulatory vectors aim to engage a strong immune response against the GBM cells. This can be achieved by expression of strong antigens on tumor cells’ surface or by the production of factors which stimulate and attract the immune cells.

4. Introduction of anti-oncogenes and tumor suppressors in cancer cells aims to decrease proliferation, stimulate differentiation or induce apoptosis by a dominant gain-of-function effect.

To achieve maximum efficiency, some approaches can be combined. For example, an oncolytic effect may accompany release of immunomodulatory proteins expressed by genes delivered with a viral vector.

### 2.1. Viral Vector Types Proposed for Gene Therapy of GBM

**Figure 1:**
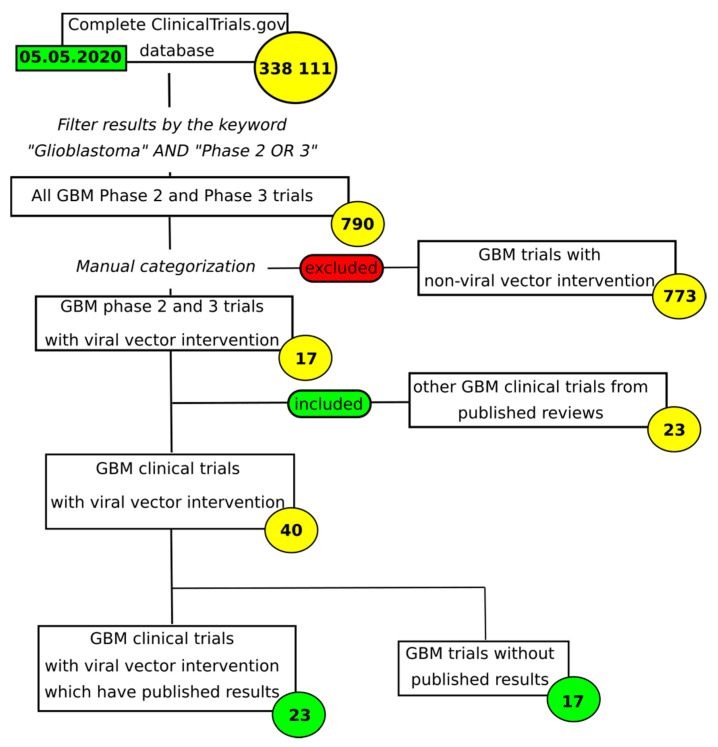
Selection and inclusion criteria for review of glioblastoma multiforme (GBM)-targeting viral vector trials.

The effectiveness of gene therapy tools is a function of virus biology, mechanism of action, specificity and replication competency. If the viral genome is partially deleted to prevent replication, this clears room for the delivery of the therapeutic genes. If, however, the virus is allowed to replicate, it will cause cytopathic effects, lysis and new virions will proceed to infect other cells. There are currently over 20 viral vectors that have been used in clinical trials for gene therapy of GBM, as summarized in Table 1. Figure 1 describes the selection criteria.

### 2.2. Adenovirus-Based Vectors

**Figure 2:**
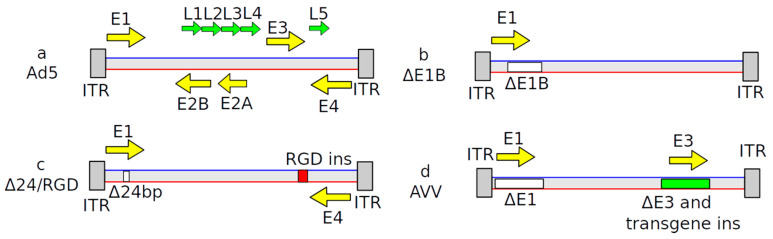
Schematic of the genome structures of adenovirus type 5 (Ad5) and Ad5-based vectors. (**a**) Wild-type Ad5 virus. Arrows indicate transcriptional units. ITR—Inverted terminal repeat. (**b**) In the ONYX-015 adenoviral vector, the *E1B* gene is deleted. (**c**) DNX-2401 adenoviral vector structure. Δ24 bp indicates 24 base pairs’ deletion in the Rb-binding domain of the E1A gene; RGD ins indicates an insertion of an additional peptide sequence in the Ad fiber-encoding part of the genome. (**d**) Adenoviral vectors, often referred as AVVs in the literature, are replication-incompetent viral particles produced by deleting *E1* and *E3* genes and inserting a desired transgene. Such vectors are widely used in experimental neuroscience for gene delivery by various groups, including ourselves [23,24,25].

Adenovirus (Ad) is a double-stranded DNA virus (Baltimore Classification class I [15]) without an envelope [16]. There are at least 57 serotypes of human Ad, Ad1–Ad57, in seven species, A–G [17]. The human Ad genome contains five early transcription units (E1A, E1B, E2, E3 and E4), four intermediate and one late transcription unit [17]. Main modification of Ad genome are shown in Figure 2. Viral entry is coxsackie-adenovirus receptor (CAR)-dependent. One of the crucial steps in the adenoviral replication cycle is interaction of the *E1A* gene product with E2F-Rb or E2F-DP1 transcription complexes to force the infected cell into the S phase since it is helps the virus to use the cellular DNA replication machinery to replicate its own genome [18]. These processes can be altered to achieve increased selectivity towards GBM and will be discussed later. Most Ad vectors originate from Ad5 (Species C). Non-replicating Ads are widely used as experimental gene delivery tools, while replicating Ads have been engineered to be tumor-specific agents. The conventional strategy to achieve replication deficiency is to delete *E1* and *E3* genes. The genomes of such vectors, after entering the target cell nucleus, remain as additional DNA elements not integrated into the chromosomes (i.e., episomal). This has major implications for their fate in the cancer, as well as in any other dividing cells, because after a few divisions, episomes which do not replicate are diluted and expression drops rapidly.

The strategies for targeting Ad vectors to GBM include (1) use of tumor-specific promoters; (2) deletion of critical viral genes which are supplied by tumor cells in trans; (3) modification of the viral capsid to enable selective entry into GBM cells.

ONYX-015 was the first oncolytic Ad vector to be described [19]. This is a recombinant selectively replication-competent chimeric Ad2 and Ad5 vector [17]. ONYX-015 lacks the *E1B* gene. The normal function of the protein encoded by *E1B* is to inactivate p53 protein in infected cells. Thus, ONYX-015 was expected to replicate only in p53-deficient cells [20], but later, it was found that ONYX replication is not, in fact, p53-dependent [21,22].

DNX2401 (Delta-24-RGD) is a recombinant serotype 5 strain Ad [26]. This oncolytic vector has two modifications in its genome that make it selectively replication-competent in cells defective in the Rb/p16 tumor suppressor pathway. The first modification is the 24-bp deletion (bp 923–946) in the Rb-binding domain of the *E1A* gene [26]. Under normal circumstances, viral E1A proteins promote cells towards a mitotic state by releasing E2F transcriptional factors from the block by Rb proteins. The unstable version of the *E1A* gene in DNX2401 cannot bind to E2F-Rb or E2F-DP1 transcription complexes and release E1A. This prevents replication in cells with a normal Rb/p16 tumor suppressor pathway. GBM often have defective Rb/p16 tumor suppressor pathways, which makes it possible for viruses to replicate selectively in GBM cells because cells are free from the Rb/p16 block anyway. Most cancer cells lack, or poorly express, CAR receptors required for adenovirus binding and internalization. To circumvent this problem, the second modification, an additional RGD peptide sequence in the HI loop of the Ad fiber, allows the virus to bind to cells expressing integrins αvβ3 and αvβ5 which are found on the surface of most cancer cells, including glioma and GBM [26,27].

DNX-2440 (Delta-24-RGDOX) is an immunomodulatory recombinant selectively replication-competent serotype 5 strain Ad-encoding OX40 ligand (OX40L) driven by the cytomegalovirus (CMV) promoter. The protein is able to activate T cells via interaction with its receptor on the surface of T lymphocytes [28,29].

AVV-CMV-HSV-tk (Ad-hCMV-TK) uses the suicide gene strategy and is a recombinant replication-defective serotype 5 Ad with *Herpes simplex virus thymidine kinase* (*HSV-tk*) gene under the transcriptional control of the CMV promoter [30]. CMV is often referred to as ubiquitously and constitutively active. However, experimental neuroscience demonstrated that this is, in fact, not the case, since CMV-bearing viral vectors effectively drive expression only in some cell types in the normal rodent brain and expression may be transient [31]. It follows that the brain cells have mechanisms to silence CMV and this may very well apply to the clones within GBM.

AVV-RSV-HSV-tk (ADV/HSV-tk) is a similar suicide gene virus but expresses *HSV-tk* under control of Rous sarcoma virus long-terminal-repeat promoter (RSV) [32]. The RSV promoter is considered a strong constitutive promoter, similar to CMV. RSV, in comparison with CMV, exhibits a lag phase prior to the onset of viral DNA replication and has a somewhat different profile of tissue-specific expression, although it is not entirely clear whether this confers an advantage in this case [33].

Ad-hCMV-Flt3L is a recombinant replication-deficient serotype 5 Ad for CMV promoter-driven expression of human fms-like tyrosine kinase 3 ligand (Flt3L). Flt3L is a hematopoietic growth factor and ligand for the Flt3 tyrosine kinase receptor, which is expressed on the surface of dendritic cells (DCs). The transgene provides an immunomodulatory effect by stimulating both the proliferation of dendritic cells and their migration to the tumor site. The vector is usually used with other conventional drugs for eliciting a stronger response to GBM via release of Flt3L from destroyed cells [34].

**Figure 3:**
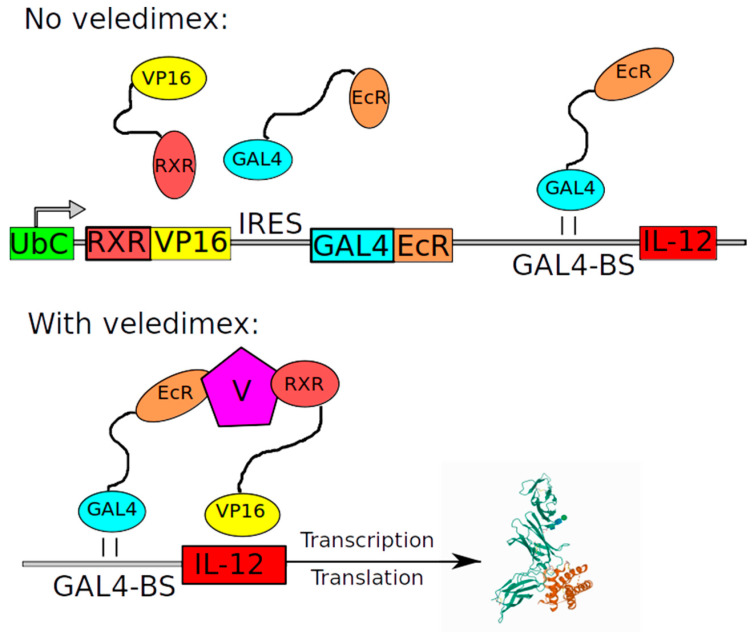
Schematic representation of *RTS* gene switch cassettes. Upon administration of veledimex, RXR-VP16 and GAL4-EcR proteins dimerize and activate transgene expression. The GAL4 domain recognizes unique specific binding sites (GAL4-BS) while VP16 acts as a powerful activation of transcription in mammalian cells. The protein 3D structure was adopted from Yoon et al. [37].

Ad-RTS-hIL12 also aims at immunomodulation. It is a recombinant replication-deficient serotype 5 Ad-encoding human pro-inflammatory interleukin-12 (IL-12: hIL indicates human origin of the gene) gene under control of RheoSwitch Therapeutic System (RTS) promoter. RTS is an artificial veledimex-inducible promoter that leads to uniform and long-term release of interleukin-12 in the tumor area after a single vector injection. This system is based on recruiting transcription factor to a synthetic promoter via Gal4–Gal4-binding site interactions [35]. The cassette consists of Gal4-EcR fusion protein sequence, internal ribosome entry site (IRES) linker and *VP16-RXR fusion protein* gene and is driven by *human ubiquitin C* gene promoter (Figure 3). Upstream, there is a customizable promoter with Gal4 binding sites to which these fusion proteins are recruited and the target gene is transcribed [35]. IL-12 activates the immune system, which may result in immune-mediated tumor cell lysis and inhibition of cancer cell proliferation [36].

Ad.hIFN-β is another immunomodulating replication-defective serotype 5 Ad-encoding human *Interferon-β* (*IFN-β*) gene under control of CMV promoter [38]. Interferon-β (IFN-β) is a pleiotropic cytokine with anti-tumor activity which demonstrated promising outcomes in some clinical trials [39]. However, overall efficacy was limited and transient mainly because of high-dose toxicity (myelosuppression, transaminitis, neurotoxicity, including seizures, etc.) [38]. To overcome this limitation, Ad.hIFN-β was developed to drive synthesis of Interferon-β in cancer cells. A schematic representation of the genome is shown in Figure 2.

VB-111 is recombinant replication-defective serotype 5 Ad-encoding Fas-TNFR-1 gene under control of pre-proendothelin-1 promoter. The promoter was chosen with the aim of achieving selectivity to endothelial cells undergoing angiogenesis. Cell apoptosis is induced when circulating TNF-α interacts with the Fas-TNFR-1 receptor [40]. The expected outcome is the prevention of vascularization and, therefore, metabolic insult to the tumor.

As mentioned above, replication-incompetent Ad vectors stay episomal in the transduced cells and are not propagated when the cell divides. This leads to a rapid dilution of the viral genomes in any dividing cells, such as GBM. In this respect, replication-competent viruses, such as ONYX-015, are different because they replicate in the affected cells. The downside of this strategy is the lack of control over the spread of the virus and infection of the healthy cells, which then, inevitably, become targets for 5FC. In addition, release of the activated, toxic products of pro-drugs non-selectively kills adjacent cells (the “bystander effect”).

### 2.3. Herpes Simplex Virus-Based Vectors

**Figure 4:**
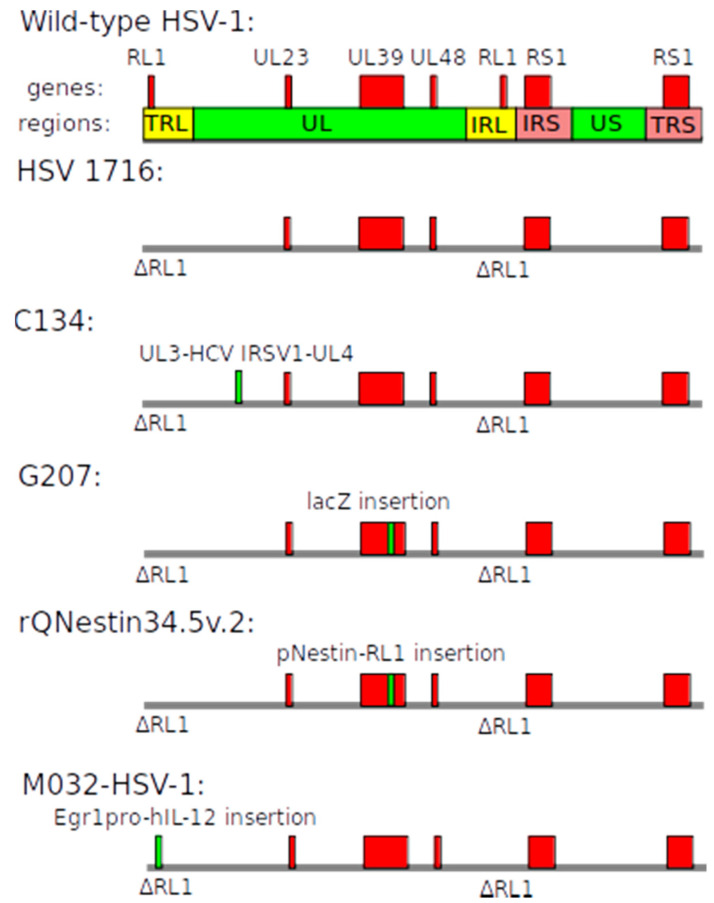
Schematic of the *Herpes simplex* virus 1 (HSV-1)-based vectors. The genome of wild-type HSV-1 can be divided into six regions which contain specific genes. Information about specific vectors is provided in the text.

*Herpes simplex* virus (HSV) is an enveloped double-stranded DNA virus (Class I according to the Baltimore classification [15]). HSV can target both dividing and non-dividing cells and has broad tropism but predominantly infects neurons. Herpes viruses are classified into subfamilies, and for gene therapy applications, HSV-1 is used. The genome of HSV-1 is ~150 kbp long and can, therefore, potentially carry a substantial payload (Figure 4). During the viral life cycle, HSV-1 remains episomal as a circular DNA molecule [41].

The RL1 gene (also known as γ34.5), one of the essential genes for replication, can be used to modulate specificity. During viral replication, the host cellular defense system typically responds with translational arrest and reduction in the global synthesis of viral and cellular proteins [42]. This process is facilitated by phosphorylation of the translation initiation factor eIF2α by protein kinase R (PKR). *RL1* gene encodes The Infected Cell Protein 34.5 (ICP34.5), also known as Neurovirulence factor ICP34.5. This multifunctional protein binds and retargets the host phosphatase PP1α to eIF2α, thus reversing the phosphorylation and the shutdown of the protein synthesis [43]. Mutated ICP34.5 is unable to counteract PKR action, which, theoretically, should protect healthy cells. Since in tumors, the PKR pathway is often inhibited, lack of ICP34.5 function does not limit viral replication and should result in selective replication of this mutated HSV-1 in such cancer cells.

The other important HSV-1 gene is UL39, which encodes the large subunit of ribonucleotide reductase, also known as ICP6. The ribonucleotide reductase complex converts ribonucleotides to deoxyribonucleotides needed for viral DNA replication. The host ribonucleotide reductase enzyme is highly active only in mitotic cells. Thus, UL39-defective HSV-1 UL39 cannot replicate efficiently in non-dividing cells [44]. Specific examples are given below.

HSV 1716 is an oncolytic recombinant replication-competent HSV-1. Deletions in both copies of the *RL1* gene (see above) were made with the aim to permit replication only in PKR-defective tumor cells [45].

C134 is an oncolytic HSV-1. In this virus, *RL1* genes are deleted and human cytomegalovirus (HCMV) *IRS1* gene was inserted between *UL3* and *UL4* genes [46]. The *IRS1* gene enhances replication in fast dividing tumor cells [46]. The exact molecular mechanism of action of IRS1 protein is still not known.

G207 is an oncolytic recombinant replication-competent HSV-1 which has two modifications to increase specificity towards GBM cells: deletions in both copies of the *RL1* gene to target PKR-defective cancer cells and disruption of *UL39* gene to eliminate the possibility to replicate in non-dividing normal cells. During the lytic phase, the vector causes direct cytopathic effect and indirect T cell-mediated cell death [47].

rQNestin34.5v.2 is a recombinant HSV-1 also devoid of *UL39* and all *RL1* genes. Lack of *RL1* gene should limit replication in normal cells via the mechanism explained above. Instead, this vector carries one copy of *RL1* gene under transcriptional control of the nestin promoter, which is frequently upregulated in gliomas [48]. Thus, nestin promoter is expected to drive expression of functional ICP34.5 selectively in glioma cells, resulting in a cytopathic effect. It is worth noting that the selectivity of this promoter is not widely known and that nestin is also expressed in normal brain cells [49].

M032-HSV-1 is a combined (oncolytic and immunomodulatory) replication-competent HSV-1. The virus has deletions of both copies of the *R1* (*γ34.5*) gene and inserted *interleukin-12* (*IL-12*) gene [50]. Deletions limit replication to PKR-defective tumor cells. In addition, interleukin-12 promotes an immune response against surviving tumor cells and decreases angiogenesis.

### 2.4. Vectors Based on other Viral Backgrounds

Pelareorep (Reolysin) is a human wild-type reovirus [51,52]. Reovirus is a non-enveloped double-stranded RNA virus (Class III according to the Baltimore Classification [15]). It causes mild infections in humans—for instance, gastroenteritis. Reoviruses can be used as oncolytic agents because they replicate predominantly in cells where the Ras pathway is highly active, as is typical for many cancers [53]. Specific examples are provided in Table 1 and Table 2.

Newcastle disease virus (NDV) is a single-stranded enveloped RNA virus whose natural host is poultry. It has been shown that the virus can induce apoptosis in melanoma cultures overexpressing a protein called Livin, encoded by the BIRC7 gene. This protein belongs to a family of anti-apoptotic proteins which are commonly overexpressed by tumors and it has been demonstrated that melanoma tumor cells that do not express Livin are relatively resistant to the virus [54]. Attempts have been made to use it against GBM [54]. NDV-HUJ is a wild-type oncolytic HUJ strain of Newcastle disease virus.

ParvOryx, or H-1PV, is an oncolytic wild-type parvovirus, a small single-stranded DNA virus (Class II according to the Baltimore classification [15]) without an envelope. In nature, this is a rodent virus, but H-1PV is able to infect cells of other species, including humans. Replication of H-1PV greatly depends on the activity of the host enzymes expressed during the S-phase, making it selectively replication-competent in fast dividing cancer cells [55].

PVSRIPO is a poliovirus type 1 (Sabin type) viral vector with its cognate internal ribosome entry site (IRES) replaced with that of human rhinovirus type 2. The vector binds to CD155 (poliovirus receptor, PVR or NECL5), internalizes and eventually causes tumor cell lysis [56]. The exchange of the IRES should, in theory, restrict replication in cells of neuronal origin [56].

Toca 511 is a replicating gamma-retrovirus which carries a *yeast cytosine deaminase (CD*) gene. Administration of 5-FC leads to generation of toxic 5-FU by CD [57]. As a result, tumor cells infected by this virus should die and release 5-FU, which can cause the bystander effect [58]. The vector has specificity for replicating cells, and replication in non-malignant cells in vivo is reportedly insignificant [59].

TG6002—recombinant vaccinia viral vector, also encoding the suicide gene *CD* [60]. Vaccinia virus is a 190-kbp dsDNA-enveloped virus which causes small pox [61]. To increase safety and specificity to fast dividing cells, the *J2R* gene (encoding thymidine kinase) and the *I4L* gene (encoding the large subunit of the ribonucleotide reductase) were deleted [61].

MV-CEA is a recombinant Edmonston strain of measles virus, expressing a soluble extracellular *N*-terminal domain of human carcinoembryonic antigen (CEA) [62]. Internalization is mediated through CD46 binding, leading to formation of syncytium and cell lysis [62]. The expressed CEA is expected to stimulate the immune system to recognize and destroy targeted cells.

### 2.5. Evaluating Vector Efficacy

The main goal of patient treatment is to increase life expectancy and improve the quality of life. Unfortunately, GBMs are a very heterogeneous group of diseases. Even morphologically-similar tumors can have different driver mutations and responses to treatment, which makes it impossible to directly compare the results of clinical trials. It should be noted that regional features of healthcare systems and even personal experiences of the attending physician can introduce bias. Moreover, previous treatment changes the tumor makeup due to clonal selection, which must be taken into account.

For the purpose of this review, we have stratified studies into three types.

1. Dose-escalating studies to assess the maximum acceptable dosage of the gene therapy vector. In accordance with the possible side effects of the administration of viral vectors, these studies are not carried out on healthy volunteers.

2. Comparison of the new therapy with existing ones when used in patients with recurrent or progressive GBM. In such patients, the prior therapy has led to the emergence of resistance and more aggressive clones, thereby diminishing the potential benefit of TMZ and justifying the application of a new therapeutic regime.

3. Comparison of the new treatment with standard treatment in patients with newly diagnosed GBM. If a therapy has shown effectiveness against TMZ-resistant GBM, it is advisable to study it in new cases as an alternative (or even replacement) to standard treatment.

We also deliberately include the date on which the study record was first available on ClinicalTrials.gov [63]. This makes it possible to identify viral vectors which have been discontinued for various reasons (including insufficient efficacy) from those that are still in ongoing trials but without published results yet (Table 2).

## 3. Discussion

The search for a gene therapy solution is driven by the abysmal prognosis currently typical for GBM. As of today, many different ideas have been proposed and tested, some of which are summarized above. However, so far, no obvious breakthrough is evident.

Of the many studies listed in Table 2 and other parts of this review, we have selected two, both using Ad, which have led to interesting results and were published recently. They pursue different strategies and are interesting to compare.

Lang et al. reported the outcome of the trial of DNX 2401 (Delta-24-RGD) on 25 patients without surgical resection and 12 patients where the vector was first injected into the tumor via an implanted catheter, which was followed by surgical removal of the tumor 14 days later and multiple intramural injections of DNX 2401 [64]. Viral loads varied between cases between 10^7^ and 3 × 10^10^ viral particles (vp) in 1 mL volumes. The paper mentions that 3 × 10^10^ vp in 1 mL was the highest concentration of Ad which could be manufactured, which is close to the experience of our laboratory. In the group treated with a single intratumoral injection of DNX 2401 (no surgery), tumor reduction occurred in 18 of 25 patients (72%). The median survival time in that group was only 9.5 months, regardless of the vector dose, which does not look to be a major success; however, five patients (20%) from this group survived for more than 3 years, which is rather striking given that they were all initially enrolled as recurrent cases with previous history of drug treatments and resistance. Obviously, all patients also received therapies other than DNX 2401. Some limited spread of the vector outside of the brain was detected and anti-Ad5 antibodies appeared in a significant number of patients in both cohorts. In histological specimens, various signs of immune response and inflammatory infiltration as well as viral cell death were evident. The incidence of side effects was very high—for example, 68% experienced headaches, 32% experienced hemiparesis, and 24% convulsions—but the authors argue that they were mainly disease- and not treatment-related. Overall, the paper shows clearly that DNX 2401 can induce an oncolytic effect accompanied by an immune response. This study can, perhaps, be seen as one of the fairly successful preliminary trials which relies on the concept of conditionally replicating oncolytic viruses. From the available information, it seems that the control provided by the requirement for the defective Rb/p16 pathway, as characteristic for many tumors, is sufficiently tight, and the spread of the virus was obviously not too fast and was limited to the locality of injection, rather than becoming generalized encephalitis, which is encouraging news. It is a pity that the integrity of Rb/p16 was not assessed in the patients’ biopsies—perhaps that could help to predict the efficacy of the treatment. It would also be important to confirm directly that DNX 2401 is still able to infect the GBM cells after the tumor is given time to undergo clonal selection as it typically happens with GBM. Can GBM cells escape by downregulating the binding sites for the RGD motif, incorporated in this gene therapy agent? It will be very interesting to watch further developments in this dimension.

Recently, the results of NCT02026271 (ClinicalTrials.gov Identifier), which uses Ad–RTS–hIL-12, were published [71]. It is interesting to analyze the approach used in that study in more detail since it highlights many problems facing the field. As mentioned above, Ad–RTS–hIL-12 is a replication-incompetent AVV with a promoter, controllable by a small-molecule drug veledimex (VDX), allowing drug-induced production of interleukin-12 (IL-12) by the cells where AVV genomes are active. The study mainly focused on the demonstration of the ability to induce IL-12 production by VDX and the safety of this treatment. Patients enrolled were all already previously treated with various regimes and, obviously, represented a really tough challenge. After surgical resection of the bulk, AVVs were injected into one spot in white matter as a single injection of 50 µL containing 2 × 10^11^ viral particles, which corresponds to the titer of 10^13^ vp/mL, which our laboratory was never able to achieve and seems to be an extremely concentrated AVV stock administered in a very small volume (compare to the previously mentioned paper [70]). The drug treatment lasted for 14 days. During that period, the drug clearly induced production of IL-12, which spilled over into the systemic circulation, and various signs of inflammatory response were visible in the patients; luckily, they were easily reversible by VDX discontinuation. Interestingly, patients treated with 20 mg VDX seemed to survive better than both those treated with lower and higher doses, the latter probably being a sign of a negative effect of excessive immunostimulation. Over the 30-month observation period, 30 of 31 enrolled patients died, which can hardly be considered a therapeutic success. Nevertheless, the authors successfully demonstrated infiltration of the tumor by the immune cells, indicating that, at least mechanistically, they achieved the expected result. Considering the results of this study, as reasoned above, non-replicating AVV genomes are inevitably diluted in dividing tumor cells. Since the whole protocol lasted for 14 days, this could be the only period when there was enough active transgene in the remaining GBM cells. Unfortunately, in the paper, there is no information on the presence of the viral genomes in the post-mortem samples. This issue, i.e., survival of the transcriptionally active adenoviral genomes in the GBM, is both interesting and important but we do not have the answer yet. It would be very interesting to know whether VDX could effectively trigger a wave of IL-12 production 3–5 months after the transduction. The other question is whether the cells producing IL-12 were mainly the GBM cells or other cells in the vicinity of the injection track. Overall, this strategy is in progress and seems to critically depend on the ability to quickly destroy the infiltrating GBM cells while the AVV are still functional.

What are the limitations, and can they be overcome, at least theoretically? The first point to consider is that of infection or transduction efficiency and stability of transgene expression. Viral vectors must be able to very efficiently enter the target cells and introduce any transgene cargo into their nuclei. Viral vectors have been extensively used in biomedical research and neuroscience for the last 20 years and there is a wealth of information about many of the vectors, similar to those used in human trials. For example, the internalization mechanism of species C adenoviruses is based on their interaction with CAR and Integrin αvβ5 proteins on the surface of the target cells [90], PVSRIPO requires CD155 [56], MV-CEA cell entry is based on interaction with CD46 [62], and so forth. We argue that this makes strategies involving adenoviral and similar vectors, which require specific GBM surface proteins for entry, vulnerable to the common mechanism of tumor defense based on downregulation of the relevant proteins and consecutive clonal selection and expansion. Ad has been used in vitro by many groups, including ourselves, in experimental neuroscience for transgene expression in both neurons and glia [91,92]. In vivo, however, these vectors clearly prefer astrocytes over all other cell types in the brain [25,92], and thus, unmodified Ad cannot be seen as a universally efficient delivery tool, irrespective of the putative origin of the GBM. In some Ad-derived gene therapy vectors, such as DNX 2410, a specific modification of the fiber H-loop should enable them to bind to specific integrins expressed by many tumor cells, but this mechanism is vulnerable to downregulation of the target integrins. The obvious differences in transductional tropism between adeno- and lentiviral vectors in rodent CNS were demonstrated long ago [93]. It was noted that vesicular stomatitis virus G-protein (VSVG)-pseudotyped lentiviruses which do not utilize a specific receptor-dependent entry pathway have a much wider transduction potential. In our laboratory, VSVG-pseudotyped HIV-derived lentivirus was used to transduce six patient-derived GBM cell lines with an apparent 100% success rate (unpublished observations). We suggest that the requirement for a specific interaction partner protein on the target cells is a limitation of vectors used for gene therapy of GBM because these can be easily eliminated by selection, making tumor cells resistant. Could lentivirus be a route to explore? Another fundamental issue is the possible silencing of exogenous expression cassettes. In experimental neuroscience, this was noted a long time ago for a commonly used promoter CMV, which is incorporated in several viral vectors listed here [31,94]. The mechanisms of CMV-mediated transgene silencing are not well understood but could be based on RNA interference or methylation of the viral promoters by cell defense machinery [95,96]. Additionally, as mentioned above, replication-incompetent vectors which stay episomal fail to propagate to the progeny of the cells they invade, which means that unless the infected GBM cells die immediately, they will eliminate viral genomes by dilution after a few divisions.

The next important point is the mechanism of action of viral gene therapy. Oncolytic viruses use the natural feature of viruses to multiply and destroy cells. Obviously, such processes, if uncontrolled, will be lethal, as exemplified above by Reolysin or C134. Various mechanisms of transcriptional control are used to enable replication predominantly in fast dividing cells. However, if this strategy is really successful and, thus, leads to a powerful cytopathic effect, rapid destruction of GBM in clinical settings can cause brain edema with subsequent impairment of vital functions and even death. Specificity of viral gene therapy is a fundamental problem. For cytopathic viruses, this solely relies on the dependence of their replication on factors highly expressed by tumor cells. However, GBM cells, even within the same tumor, are heterogenous [97]. Is it even possible to find a ubiquitous driver/controller of viral replication in the pool of diverse GBM cells? At this point, such a possibility remains to be demonstrated. So far, the selectivity of the published vectors is obviously not sufficient to fully prevent destruction of normal brain cells. With some vectors, such damage can be inflicted by the conversion of pro-drugs into toxic specimens which are then released—the so-called bystander effect. This problem is particularly relevant to the brain, where elimination, dilution and biodegradation of these harmful molecules might be slower than in the periphery. An added problem introduced by replicating vectors is the release of viral particles into the bloodstream, leading to an inevitable immune response.

The success of viral gene therapy critically depends on the physical access of the virus to the GBM cells. Shall they be injected into the brain at the time of surgery or administered using some other means? It would be ideal to inject the virus into the bloodstream because it could reach all GBM cells which are spread within the parenchyma, but can this be done? Outside of the field of neuro-oncology, the best current example of an attempt to achieve generalized expression in the human brain with an i.v.-injected viral vector is Zolgensma (AVXS-101), an adeno-associated viral vector carrying the SMN1 transgene [98]. However, in humans, this virus has to be delivered before 2 years of age, when the blood–brain barrier is still not completely mature, and large doses are used, requiring administration of steroids to prevent a severe immune response [99]. This is in stark contrast with multiple studies in mice where a brain-wide expression has been achieved with some strains of adeno-associated virus injected i.v. [100]. Adeno-associated viruses are extremely small and definitely have the best chances of reaching the CNS when their concentration in the bloodstream is high enough, but they do not seem to have any tropism to GBM in addition to the fact that the adult human BBB is probably completely impermeable to them. Moreover, after a single application into the bloodstream, a strong antibody response is inevitable, making this a “single shot only” strategy. It is therefore unlikely that we will see successful targeting of disseminated GBM with any type of currently available viral vector applied via the bloodstream.

To summarize, the attempts to develop an efficient gene therapy for GBM with viral vectors face the following fundamental problems.

(a) Vectors relying on a specific mechanism of internalization are unlikely to be successful because of the extreme instability of GBM genomes, the multitude of clones in the same tumor and the ease of clonal selection of resistant cells to which the virus will have no access. It follows that using less specific mechanisms of viral entry might be a winning strategy.

(b) GBM cells divide, and some do it at a very high pace. In such cells, non-integrating viral genomes will be rapidly diluted and probably become inefficient, unless they cause immediate death of the cell. The ability to silence transgenes adds to this problem. The only way to ensure downwards transmission of the transgene is the use of integrating vectors, such as lentiviruses.

(c) Specificity of the effect is one of the key requirements and we have listed, above, some of the strategies used to limit the impact to GBM cells vs. the rest of the brain. So far, many of these strategies have been demonstrated to work in vitro and sometimes even in GBM-bearing mice in vivo. Whether a sufficiently reliable and universal strategy can be found for clinical application remains to be seen. We hypothesize that one avenue to explore is to try to suppress the mitotic apparatus, since healthy cells in the postnatal human brain rarely or never divide.

(d) Injection in the bloodstream is unlikely to be successful. We are therefore left with a necessity to infiltrate with viral gene therapeutics the areas of the putative GBM growth during the debulking surgery or, possibly, by stereotaxis at a later stage.

We hope that this review will allow readers to get a feel for the current options for the viral gene therapy of GBM and initiate a discussion about its future directions. We suggest that a more plausible strategy might be to focus on viruses which enter via a non-selective, ubiquitous mechanism. We hypothesize that it might be possible to irreversibly block processes critical for dividing tumor cells which are dispensable for quiescent healthy brain cells. Mitosis is a highly specialized stage of a cell’s life and depends on a range of proteins which are expressed in non-dividing cells at low levels. This idea may be illustrated by the current attempts to target, for example, cyclin-dependent kinases with inhibitors. The key difference is that the peripheral cells—for example, in the bone marrow—should not be affected and inhibited by a virus which is delivered into the brain parenchyma. Hence, the issue of systemic toxicity could become less critical.

As stated in the beginning, this review reflects the view of the experimentalist neuroscientists and, hopefully, might stimulate a discussion leading to new discoveries in the field of neuro-oncology.

## 4. Conclusions

Viral gene therapy of GBM is a promising field but several major hurdles need to be overcome for it to become an accepted part of the currently available portfolio of therapeutic interventions. As yet, some potentially encouraging results have been obtained with a conditionally replicating oncolytic Ad, but the fundamental challenge of tumor resistance via downregulation of the proteins, critical for viral proliferation remains to be overcome. Obviously not all the options have been yet explored and we hope to see new types of vectors entering clinical trials in years to come.

## Figures and Tables

**Table 1:** Comparison of key features of viral vectors proposed for treatment of GBM.

Name	Structure of Vector	Mechanism of Action	Specificity	Replication Competent
DNX2401	Ad5	Lytic viral cycle in targeted cells	Replicate in cells defective in the Rb/p16 tumor suppressor pathway and expressing integrins αvβ3 and αvβ5	±
DNX2440	Ad5	Lytic viral cycle in targeted cells and immunomodulatory effect	Replicate in cells defective in the Rb/p16 tumor suppressor pathway and expressing integrins αvβ3 and αvβ5	±
ONYX-015	chimeric Ad2 and Ad5	Lytic viral cycle in targeted cells	Replicate in tumor cells with altered p53 pathway	±
Ad-hCMV-TK	Ad5	Converts harmless ganciclovir to toxic product in transduced cells	Transduce CAR-expressing cells. CMV-dependent expression mechanism	−
ADV/HSV-tk	Ad5	Converts harmless ganciclovir to toxic product in transduced cells	Transduce CAR-expressing cells. RSV-dependent expression mechanism	−
Ad-hCMV-Flt3L	Ad5	Immunomodulatory effect by stimulating both the proliferation of dendritic cells (DCs) and their migration to the tumor site	Transduce CAR expressing cells	−
Ad-RTS-hIL12	Ad5	Immunomodulatory effect by activation of immune system via IL-12 release	Transduce CAR-expressing cells	−
Ad.hIFN-β	Ad5	Immunomodulatory effect by activation of immune system via human Interferon-β release	Transduce CAR-expressing cells	−
VB-111	Ad5	Decrease excessive angiogenesis via inhibition of endothelial cells	Transduce CAR-expressing cells, promotor initializes transcription only in endothelial cells undergoing angiogenesis	−
HSV 1716	HSV-1	Lytic viral cycle in targeted cells and indirect T cell-mediated cell death	Replication in PKR-deficient cells	±
G207	HSV-1	Lytic viral cycle in targeted cells and indirect T cell-mediated cell death	Replication in PKR-deficient and fast dividing cells	±
C134	HSV-1	Lytic viral cycle in targeted cells and indirect T cell-mediated cell death	Replication in PKR-deficient and fast dividing cells	±
rQNestin34.5v.2	HSV-1	Lytic viral cycle in targeted cells and indirect T-cell mediated cell death	Replication in PKR-deficient, Nestin-positive and fast dividing cells	±
M032-HSV-1	HSV-1	Lytic viral cycle in targeted cells, indirect T-cell mediated cell death and immune system stimulation via IL12 release	Replication in PKR-defective and fast dividing cells	±
Pelareorep (Reolysin)	Wild-type reovirus	Lytic viral cycle in targeted cells	Replication in ras-positive cells	+
ParvOryx	Wild-type parvovirus	Lytic viral cycle in targeted cells	Replication in fast dividing cells	+
NDV-HUJ	Wild-type HUJ strain of Newcastle disease virus	Livin-mediated apoptosis	Replication in fast dividing cells, apoptosis of livin-positive cells	+
PVSRIPO	Recombinant poliovirus type 1	Lytic viral cycle in targeted cells	Replication restricted to CD155-expressing non-neuronal cells	+
Toca 511	Recombinant Gammaretrovirus	CD-mediated prodrug conversion to cytotoxic drug in transduced cells	Replication in fast dividing cells	+
TG6002	Recombinant vaccinia virus	Lytic viral cycle in targeted cells, CD-mediated prodrug conversion	Replication in cells expressing ribonucleotide reductase	+
MV-CEA	Recombinant measles virus	Lytic viral cycle in targeted cells	Transduce CD46-expressing cells	+

In relation to the ability to replicate, + denotes replication competent vectors, − stands for replication incompetent ones and ± for conditionally replication competent vectors. CAR-chimeric antigen receptor; CMV–cytomegalovirus; RSV-rous sarcoma virus; PKR-protein kinase R; HUJ-Hebrew University, Jerusalem; CD-cytosine deaminase.

**Table 2:** Clinical trials using viral vectors.

Vector	A Unique Identification Code Given to Clinical Study Registered on ClinicalTrials.gov	Study Date	Study Type (Safety/Trials in Recurrent GBM/Trials in Newly Diagnosed GBM)	Results/Comments
DNX2401	NCT00805376	2008	Dose-escalation study in recurrent GBM	Reported in 2018: DNX-2401 is safe, improves clinical outcome. Post-treatment histology examination of biopsy revealed sites of necrosis in GBM [64].
-	NCT01582516	2012	Dose-escalation study in recurrent GBM	No posted results.
-	NCT01956734	2013	Safety and efficacy study in recurrent GBM DNX2401 + TMZ vs. TMZ alone	Reported in 2017: The safety objective of the trial was achieved with no severe toxicities related to DNX-2401 [65].
-	NCT02197169	2014	Safety and efficacy study in recurrent GBM, DNX2401 + IFN vs. DMX2401 alone	Reported in 2017: DNX-2401 was well tolerated as monotherapy. The addition of interferon did not improve survival [66].
-	NCT02798406	2016	Safety and efficacy study in recurrent GBM, DNX2401 + pembrolizumab	No posted results.
DNX2440	NCT03714334	2018	Safety and efficacy study in recurrent GBM, DNX2440 alone	No posted results.
ONYX-015	Was not registered at ClinicalTrials.gov	-	Dose-escalation study	Reported in 2004: None of the 24 patients experienced serious adverse events related to ONYX-015 [67].
ADV/HSV-tk	NCT00589875	2008	Study of AdV-tk + valacyclovir Gene therapy in combination with standard radiation therapy for malignant glioma	Reported in 2016: Addition of ADV/HSV-tk to SoC improves outcome [68].
-	NCT00870181	2009	Safety and efficacy of intravenous-administered ADV/HSV-tk in recurrent GBM vs. surgery or systemic chemotherapy or palliative care	Reported in 2016: ADV/HSV-tk is safe and can provide benefits [69].
-	NCT03603405	2018	Safety and efficacy study of standard treatment + ADV/HSV-tk in newly diagnosed GBM	No results posted.
-	NCT03596086	2018	Safety and efficacy of ADV/HSV-tk in recurrent GBM	No results posted.
Ad-hCMV-Flt3L + 4. Ad-hCMV-TK (combination)	NCT01811992	2013	Dose-escalation study in newly diagnosed GBM + standard treatment	Reported in 2019: Examination of tumor samples reveals increase in the infiltration of inflammatory cells. Preliminary data suggest that virotherapy can improve outcomes [70].
Ad-RTS-hIL12	NCT02026271	2014	Safety and tolerability of a single tumor injection of Ad-RTS-hIL-12 given with oral veledimex (the activator of RTS promoter) in patients with recurrent or progressive GBM	Reported in 2019: The clinical trial demonstrated tolerability of veledimex-induced hIL-12 expression [71].
-	NCT04006119	2019	Safety and efficacy of intratumoral Ad-RTS-hIL-12 and oral veledimex in combination with cemiplimab-rwlc in patients with recurrent or progressive GBM	No results posted.
Ad.hIFN-β	Was not registered	-	Dose-escalation study	Reported in 2008: The most common adverse events were considered by the investigator as being unrelated to treatment [38].
VB-111	NCT01260506	2010	Dose-escalation study of VB-111 in combination with bevacizumab in recurrent GBM.	Reported in 2013: VB-111 was safe and well tolerated in patients with recurrent GBM with repeat doses of up to 1 × 10^13^ VPs. Tumor responses were seen [72].
-	NCT02511405	2015	Comparison of VB-111 plus bevacizumab to bevacizumab in patients with recurrent GBM	Reported in 2020: Upfront concomitant administration of VB-111 and bevacizumab failed to improve outcomes [73].
HSV 1716	Was not registered	-	Safety and feasibility of intratumoral administration of HSV1716	Reported in 2000: HSV1716 is safe when injected into sites around the post-resection tumor cavity [74].
-	Was not registered	-	Efficacy of HSV1716	Reported in 2002: HSV1716 replicates in HGG without causing toxicity [75].
-	Was not registered	-	Efficacy of HSV1716	Reported in 2004:Study demonstrates that HSV1716 injections can provide benefits [76].
G207	Was not registered	-	Dose-escalation study	Reported in 2000: No viral-related toxicity; evidence of antitumor activity. While adverse events were noted in some patients, no toxicity or serious adverse events could unequivocally be ascribed to G207 [77].
-	NCT00028158	2001	Dose-escalation study. Doses 1E9, 3E9 and 1E10 pfu were tested	Reported in 2009:No encephalitis; evidence of antitumor activity and viral replication [78].
-	NCT00157703	2005	De-escalation study. First patients received the highest dose (1E10 pfu). and if excessive toxicity had occurred, the dose would be reduced for the following patients	As reported in 2014: Treatment was well tolerated with signs of improving outcomes [79].
C134	NCT03657576	2018	Dose-escalation study in recurrent/progressive GBM, anaplastic astrocytoma, or gliosarcoma	No results posted.
rQNestin34.5v.2	NCT03152318	2017	Dose-escalation study of in patients with recurrent GBM	No results posted.
M032-HSV-1	NCT02062827	2014	Dose escalation in recurrent/progressive GBM, anaplastic astrocytoma or gliosarcoma	No results posted.
Pelareorep (Reolysin)	NCT02444546	2015	Dose-escalation study of Pelareorep in combination with sargramostim in recurrent/progressive GBM	No results posted.
-	NCT00528684	2007	Dose-escalation study of Pelareorep in recurrent GBM	Reported in 2008: The intratumoral administration of the genetically unmodified reovirus was well tolerated using these doses and schedule in patients with recurrent GBM [80].
ParvOryx	NCT01301430	2011	Dose-escalation study of ParvOryx in patients with progressive or recurrent GBM	Reported in 2012 and 2017: No dose-limiting toxicity was reported but clinical response did not depend on the dose or mode of ParvOryx administration. No statistical confirmation of efficacy [81,82].
NDV-HUJ	Was not registered	-	Dose-escalation study of NDV-HUJ	Reported in 2006: Toxicity was minimal with Grade I/II constitutional fever being seen in five patients. Maximum tolerated dose was not achieved [83].
-	NCT01174537	2010	Safety and efficacy of single dose intravenously administered	No results posted.
PVSRIPO	NCT02986178	2016	Safety and efficacy of single dose PVSRIPO administered intratumorally in patients with recurrent GBM	No results posted.
-	NCT03973879	2019	Safety and efficacy of single dose PVSRIPO administered intratumorally with atezolizumab treatment in patients with recurrent GBM	Withdrawn.
-	NCT01491893	2011	Dose-escalation study of PVSRIPO administered intratumorally in patients with recurrent GBM	Reported in 2018: Intratumoral infusion of PVSRIPO in patients with recurrent WHO grade IV malignant glioma confirmed the absence of neurovirulent potential [84].
Toca 511	NCT04105374	2019	Toca 511, Toca FC and standard of care vs. standard of care in newly diagnosed GBM	Withdrawn.
-	NCT02414165	2015	Toca 511/Toca FC vs. Lomustine, Temozolomide, or Bevacizumab in recurrent GBM	Reported in 2020: administration of Toca 511 and Toca FC, compared with SoC, did not improve overall survival (11.10 months vs. 12.22 months, respectively) or other end points [85].
-	NCT01470794	2011	Dose-escalation study of Toca 511/Toca FC administered by injections into resection cavity wall in patients with recurrent GBM	Reported in 2016, 2016, 2018: Toca 511/Toca FC is safe and can provide durable complete response in some patients [86,87,88].
-	NCT01156584	2010	Dose-escalation study of Toca 511/Toca FC administered by intratumoral injections in patients with recurrent GBM	Reported in 2015, 2016:Safe and well tolerated [87,88,89].
-	NCT01985256	2013	Dose-escalation study of Toca 511/Toca FC administered by intravenously in patients with recurrent GBM	Reported in 2016: Injections were well tolerated [87].
TG6002	NCT03294486	2017	Dose-escalation study of TG6002 in patients with recurrent GBM	No results posted.
MV-CEA	NCT00390299	2006	Dose-escalation study of MV-CEA in patients with recurrent GBM	No results posted.

GBM—glioblastoma multiforme; TMZ—temozolomide; IFN—interferon; SoC—standard of care; RTS—RheoSwitch Therapeutic System; VPs—vector particles; HGG—high grade glioma; pfu—plaque forming unit; WHO—world health organization.
